# Association of Mediterranean Diet Adherence With Chronic Constipation and Chronic Diarrhea: Findings From NHANES

**DOI:** 10.1002/fsn3.70809

**Published:** 2025-08-13

**Authors:** Cheng Xu, Yu‐han Lin, Xing‐chi Yu, Gai‐bo Huang, Jing‐yi Hu, Hong Shen, Chong‐chao Li

**Affiliations:** ^1^ The First Clinical College Nanjing University of Chinese Medicine Nanjing China; ^2^ Institute of Literature in Chinese Medicine Nanjing University of Chinese Medicine Nanjing China; ^3^ Affiliated Hospital of Nanjing University of Chinese Medicine Nanjing China; ^4^ Jiangsu Provincial Research Institute of Chinese Medicine Schools Nanjing University of Chinese Medicine Nanjing China

**Keywords:** chronic constipation, chronic diarrhea, cross‐sectional study, mediterranean diet, NHANES

## Abstract

This study investigated the association of Mediterranean diet (MED) adherence with chronic constipation and chronic diarrhea, using a nationally representative sample of U.S. adults. This cross‐sectional study analyzed 11,612 participants from the 2005 to 2010 National Health and Nutrition Examination Survey (NHANES). Adherence to MED was assessed through the alternative Mediterranean diet (aMED) index. Bowel habits were categorized using the Bristol Stool Form Scale (BSFS), with chronic constipation defined as BSFS types 1–2, and chronic diarrhea as types 6–7, based on the usual or most common stool type reported by participants. Multivariate logistic regression models and subgroup analyses were employed to assess associations of MED adherence with chronic constipation and chronic diarrhea. Restricted cubic spline analyses were conducted to explore potential nonlinear relationships. Higher aMED scores were associated with a lower likelihood of chronic constipation (OR: 0.82, 95% CI: 0.75, 0.89). Participants in the highest aMED category had 43% lower odds of chronic constipation compared to those in the lowest category (OR: 0.57, 95% CI: 0.43, 0.75). Subgroup analysis indicated that this association was strongest among obese individuals (BMI ≥ 30 kg/m^2^) (OR: 0.69, 95% CI: 0.58, 0.82). No significant associations were observed between aMED scores and chronic diarrhea in the fully adjusted model. Adherence to the Mediterranean diet was significantly associated with lower prevalence of chronic constipation but showed no significant relationship with chronic diarrhea in the U.S. population. Further longitudinal research is needed to clarify causality and explore population‐specific effects.

## Graphical abstract

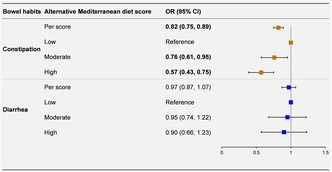

## 1 Introduction

The rapid evolution of dietary patterns and lifestyle factors has been closely associated with the increasing global prevalence of gastrointestinal disorders. Within this spectrum, chronic constipation and chronic diarrhea are the most frequently reported functional gastrointestinal conditions. Epidemiological studies demonstrate that chronic diarrhea affects between 11% and 30% of the U.S. population, while the worldwide prevalence of chronic constipation approaches 14% (Black and Ford 2018; Singh et al. 2018). Given the expanding public health impact of these conditions, the identification of effective and sustainable strategies for prevention and management has become an imperative healthcare challenge.

The Mediterranean diet (MED) has gained recognition as a potentially effective dietary intervention for improving gastrointestinal health outcomes (Martinez‐Lacoba et al. 2018). This dietary pattern, extensively studied for its health benefits, emphasizes high consumption of plant‐based foods including fruits, vegetables, whole grains, legumes, and nuts, with olive oil serving as the principal source of dietary fat (Guasch‐Ferré and Willett 2021; Sofi et al. 2008). The therapeutic potential of MED may be attributed to its rich composition of bioactive compounds, particularly polyphenols, which demonstrate anti‐inflammatory properties through modulation of inflammatory pathways (Gurău et al. 2018; Randeni et al. 2024). Furthermore, MED appears to beneficially modulate gut microbiota composition, promoting the growth of bacterial species that produce short‐chain fatty acids (SCFAs), which are crucial for maintaining intestinal barrier function and epithelial homeostasis (Barber et al. 2023; Seethaler et al. 2022).

Although existing research suggests that adherence to MED may offer gastrointestinal health benefits, evidence from large, population‐based studies remains limited. This study seeks to address this gap by exploring the association between MED adherence and chronic constipation and chronic diarrhea, using data from the National Health and Nutrition Examination Surveys (NHANES). This study aims to provide robust epidemiological evidence regarding the potential role of MED in the prevention and clinical management of gastrointestinal disorders.

## 2 Methods

### 2.1 Survey Description

This study utilized data from the NHANES, a comprehensive population‐based survey conducted by the National Center for Health Statistics (NCHS). NHANES employs a rigorous protocol to evaluate the health and nutritional status of non‐institutionalized U.S. civilians, incorporating standardized physical examinations, laboratory tests, and structured interviews. This survey utilizes a sophisticated stratified, multistage probability sampling design to generate nationally representative estimates of health parameters (Ogden et al. 2012).

All participants provided informed consent, which was approved by the NCHS Research Ethics Review Board. Detailed methodologies and datasets from NHANES are publicly available at www.cdc.gov/nchs/nhanes/.

### 2.2 Study Population

**FIGURE 1:**
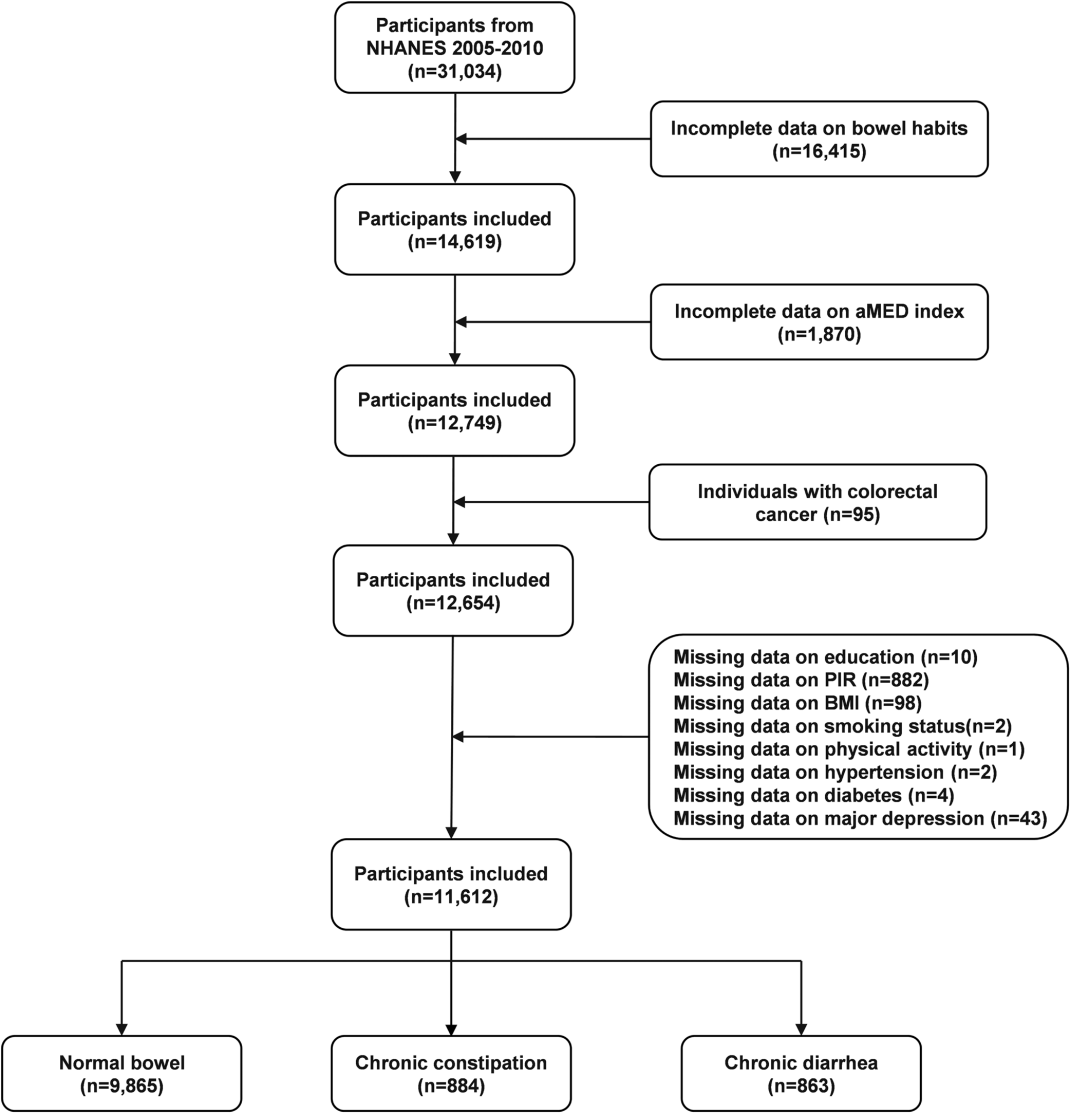
Flow chart of participants selection. aMED, alternative Mediterranean diet; BMI, body mass index; NHANES, National Health and Nutrition Examination Survey; PIR, poverty income ratio.

The analysis included data from three consecutive NHANES cycles (2005–2010), as data related to bowel health were only available during this period. The study population was limited to participants aged 20 years or older with complete datasets encompassing bowel health status, alternative Mediterranean diet (aMED) index, and relevant covariates. Exclusion criteria involved participants with incomplete bowel health records, insufficient dietary data for aMED index calculation, documented colorectal cancer diagnoses, or missing covariate information. After applying these selection criteria, the final analytical sample consisted of 11,612 participants, comprising individuals with normal bowel habits (*n* = 9865), chronic constipation (*n* = 884), and chronic diarrhea (*n* = 863) (Figure 1).

### 2.3 Definition of MED Adherence and Abnormal Bowel Habits

Dietary assessment was collected through two standardized 24‐h dietary recall interviews from the NHANES database. The initial in‐person interview was conducted during the Mobile Examination Center (MEC) visit, with a subsequent telephone‐administered recall completed within 10 days. Mean nutrient intakes were calculated from both recalls to represent habitual consumption patterns. To evaluate MED adherence, dietary data were aligned with the United States Department of Agriculture (USDA) Food Patterns Equivalents Database, enabling systematic conversion of reported foods into standardized dietary components.

Adherence to MED was evaluated via the aMED index, which encompasses the consumption levels of various food groups, including fruits, vegetables (excluding potatoes), whole grains, legumes, nuts, fish, red and processed meats, the monounsaturated fatty acids (MUFA)/saturated fatty acids (SFA) ratio, and alcohol intake (Table S1). For each dietary component, a score of 1 was allocated if the intake was above the median, except for red/processed meat and alcohol. Specifically, a point was awarded for red/processed meat intake below the median and for moderate alcohol consumption (10–25 g/day for men and 5–15 g/day for women). The total aMED score spanned from 0 to 9, with higher values indicating stronger adherence to the Mediterranean dietary pattern (Liang et al. 2022).

Bowel habits were evaluated utilizing the Bristol Stool Form Scale (BSFS). Participants were presented with a visual depiction of the scale, which encompasses seven stool types (1–7), and were asked to identify the type that most closely resembled their typical stool consistency. Chronic constipation was defined by stool type 1 (separate hard lumps, like nuts) or type 2 (sausage‐like but lumpy), while chronic diarrhea corresponded to type 6 (fluffy pieces with ragged edges, a mushy stool) or type 7 (watery, no solid pieces). Participants reporting stool types 3, 4, or 5 were classified as having normal bowel habits. (Ballou et al. 2019; Sommers et al. 2019).

### 2.4 Assessment of Covariates

Relevant sociodemographic characteristics, health behaviors, and medical conditions were included as covariates. Sociodemographic data included age, gender, race, education level, and poverty income ratio (PIR). Smoking status was classified as never, former, or current. Self‐reported physical activity levels were stratified into inactive, moderate, and vigorous categories. Body mass index (BMI), computed as weight divided by the square of height, was determined during physical examinations and divided into normal weight (< 25), overweight (≥ 25 and < 30), and obese (≥ 30) (Chen et al. 2019). Hypertension was identified based on self‐reported diagnosis, antihypertensive medication use, or elevated blood pressure measurements (systolic blood pressure ≥ 130 mmHg or diastolic blood pressure ≥ 80 mmHg). (Whelton et al. 2018). Diabetes was determined through self‐reported diagnosis, insulin or glucose‐lowering drug use, or specific glycemic thresholds (fasting plasma glucose ≥ 126 mg/dL, 2‐h glucose tolerance test ≥ 200 mg/dL, or glycated hemoglobin A1c ≥ 6.5%) (Menke et al. 2015). Major depression was evaluated using the Patient Health Questionnaire‐9, with scores of 10 or higher indicating the presence of major depression (Kroenke et al. 2001).

### 2.5 Statistical Analysis

In accordance with the NHANES analytical guidelines, sampling weights were incorporated into all analyses to account for the complex survey design. Baseline characteristics were categorized based on bowel habits status. Continuous variables were reported as weighted means along with 95% confidence intervals (CIs), whereas categorical variables were described using weighted percentages and 95% CIs. Weighted linear regression was utilized to evaluate differences in continuous variables among groups, and weighted Chi‐square tests were employed for categorical variables.

Multivariate logistic regression models were employed to calculate odds ratios (ORs) and 95% CIs to examine the associations between the aMED score and chronic constipation or chronic diarrhea. Three models were constructed: Model 1 was unadjusted; Model 2 accounted for age, gender, and race; and Model 3 further adjusted for education, PIR, BMI, smoking status, physical activity, hypertension, diabetes, and major depression. Sensitivity analyses were conducted by categorizing aMED scores into tertiles to assess the robustness of these associations.

To investigate potential non‐linear relationships between the aMED score and chronic constipation or chronic diarrhea, restricted cubic spline (RCS) analyses were conducted. Knots were positioned at the 10th, 50th, and 90th percentiles of the aMED score distribution. Subgroup analyses and interaction tests were performed based on age, gender, PIR, BMI, hypertension, diabetes, and major depression to assess potential effect modifications.

All analyses were conducted using R (http://www.R‐project.org; version 4.4.0). Statistical significance was determined using a two‐sided *p* value of less than 0.05.

## 3 Results

### 3.1 Baseline Characteristics

Baseline characteristics of the study participants are summarized in Table 1. The bowel habits analysis included 11,612 participants, of whom 9865 reported normal bowel habits, 884 had chronic constipation, and 863 experienced chronic diarrhea. Compared with those having normal bowel habits, individuals with chronic constipation were more likely to be female and non‐Hispanic Black, and they exhibited lower levels of educational attainment, PIR, BMI, and physical activity, as well as a higher prevalence of major depression. In contrast, **compared to those with normal bowel habits,** participants with chronic diarrhea tended to be older, female, and had lower educational attainment, PIR, and physical activity levels, higher BMI, increased smoking rates, and higher rates of diabetes and major depression. The chronic constipation group had significantly lower aMED scores than the normal bowel group, whereas no significant difference in aMED scores was detected between the chronic diarrhea and normal bowel groups.

**TABLE 1:** Basic characteristics of the study population.

Characteristics	Bowel habits				
	Normal bowel (*n* = 9865)	Chronic constipation (*n* = 884)	*p*	Chronic diarrhea (*n* = 863)	*p*
Age (years)	46.51 (45.75,47.26)	44.84 (43.43,46.25)	0.032	49.56 (47.97,51.16)	< 0.001
Age category (%)			0.075		0.003
20–39	37.78 (35.79,39.80)	42.73 (38.48,47.10)		30.06 (25.67,34.84)	
40–59	39.09 (37.80,40.40)	35.55 (31.05,40.31)		41.30 (36.20,46.58)	
≥ 60	23.13 (21.42,24.94)	21.72 (18.66,25.13)		28.65 (24.01,33.77)	
Gender (%)			< 0.001		< 0.001
Male	49.59 (48.40,50.78)	27.43 (23.07,32.26)		40.83 (36.59,45.20)	
Female	50.41 (49.22,51.60)	72.57 (67.74,76.93)		59.17 (54.80,63.41)	
Race (%)			< 0.001		0.130
Mexican American	7.44 (5.90,9.33)	10.31 (7.73,13.64)		8.59 (5.62,12.92)	
Other Hispanic	3.91 (2.87,5.31)	4.55 (2.89,7.09)		5.10 (3.29,7.84)	
Non‐Hispanic White	73.35 (69.70,76.71)	64.23 (57.16,70.73)		69.12 (62.45,75.08)	
Non‐Hispanic Black	10.15 (8.45,12.15)	15.66 (11.73,20.61)		12.62 (9.80,16.10)	
Other Race	5.15 (4.37,6.05)	5.24 (2.98,9.07)		4.56 (2.77,7.43)	
Education (%)			< 0.001		< 0.001
Less than high school	15.99 (14.46,17.66)	23.35 (19.67,27.48)		23.58 (19.83,27.78)	
High school	23.57 (22.13,25.07)	28.91 (24.46,33.81)		25.63 (21.69,30.01)	
More than high school	60.44 (57.93,62.89)	47.74 (42.31,53.22)		50.80 (45.33,56.25)	
PIR(%)			< 0.001		0.005
< 1.3	18.24 (16.58,20.03)	25.46 (21.17,30.29)		24.35 (20.07,29.21)	
1.3 to < 3.5	35.27 (33.15,37.46)	40.48 (35.84,45.29)		35.56 (31.37,39.98)	
≥ 3.5	46.48 (43.65,49.33)	34.06 (29.71,38.69)		40.09 (34.44,46.02)	
BMI (%)			< 0.001		< 0.001
< 25	30.67 (28.80,32.61)	39.13 (34.23,44.27)		24.62 (20.27,29.56)	
25 to < 30	33.87 (32.22,35.57)	33.67 (29.64,37.95)		29.99 (25.51,34.88)	
≥ 30	35.46 (33.63,37.33)	27.20 (23.00,31.85)		45.39 (41.38,49.47)	
Smoking (%)			0.050		0.012
Never smoker	53.48 (51.48,55.46)	58.80 (54.00,63.44)		45.47 (39.91,51.14)	
Former smoker	25.21 (23.61,26.89)	20.77 (16.98,25.14)		27.54 (23.17,32.39)	
Current smoker	21.31 (19.94,22.74)	20.44 (16.74,24.70)		26.99 (22.18,32.41)	
Physical activity (%)			0.004		0.021
Inactive	45.63 (43.43,47.84)	52.80 (47.85,57.69)		51.62 (45.70,57.50)	
Moderate	27.03 (25.51,28.60)	27.11 (22.66,32.07)		27.97 (23.41,33.04)	
Vigorous	27.35 (25.67,29.09)	20.10 (16.43,24.34)		20.40 (16.20,25.37)	
Hypertension (%)			0.055		0.065
No	51.84 (49.93,53.74)	56.14 (51.35,60.81)		47.67 (43.04,52.34)	
Yes	48.16 (46.26,50.07)	43.86 (39.19,48.65)		52.33 (47.66,56.96)	
Diabetes (%)			0.222		< 0.001
No	89.21 (88.21,90.14)	90.61 (88.39,92.44)		83.51 (79.89,86.59)	
Yes	10.79 (9.86,11.79)	9.39 (7.56,11.61)		16.49 (13.41,20.11)	
Major depression (%)			< 0.001		< 0.001
No	93.78 (92.86,94.59)	86.96 (82.71,90.28)		84.26 (79.33,88.19)	
Yes	6.22 (5.41,7.14)	13.04 (9.72,17.29)		15.74 (11.81,20.67)	
aMED score	3.53 (3.47,3.59)	3.19 (3.04,3.34)	< 0.001	3.37 (3.20,3.54)	0.056

Abbreviations: aMED, alternative Mediterranean diet; BMI, body mass index; PIR, poverty income ratio.

### 3.2 Association Between MED Adherence and Chronic Constipation

**FIGURE 2:**
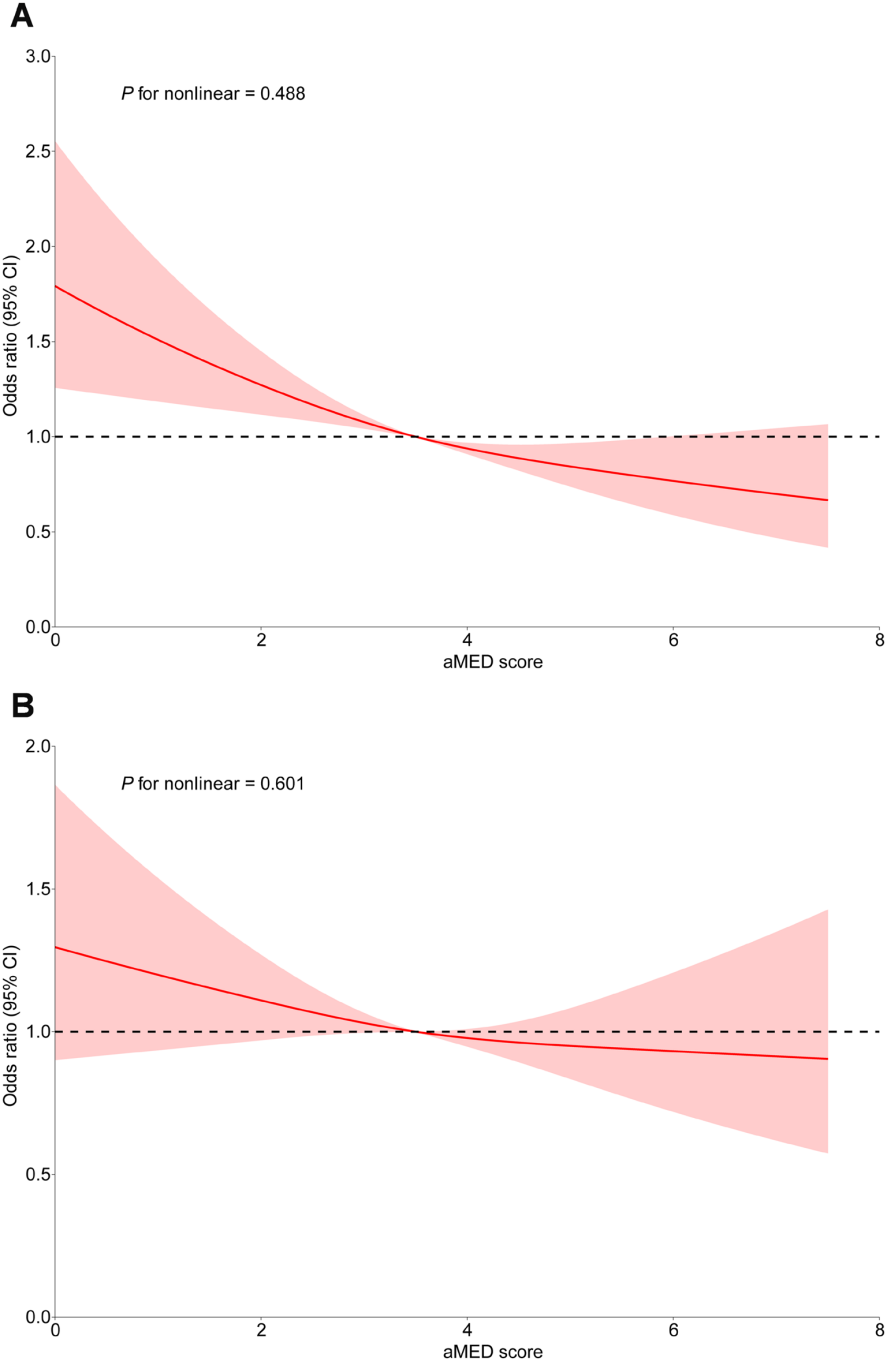
Restricted cubic spline plots of the association between aMED score and abnormal bowel habits. (A) Chronic constipation; (B) Chronic diarrhea. The solid red lines correspond to the central estimates, and the red‐shaded regions indicate the 95% confidence intervals. The model was adjusted for age, gender, race, education, PIR, BMI, smoking, physical activity, hypertension, diabetes, and major depression. aMED, alternative Mediterranean diet.

Table 2 demonstrates an inverse association between aMED scores chronic constipation across all models. Specifically, in the fully adjusted model, elevated aMED scores corresponded to reduced odds of chronic constipation (OR: 0.82, 95% CI: 0.75, 0.89). When stratified into categories, individuals in the highest aMED scores category exhibited a 43% reduced lower likelihood of chronic constipation compared to those in the lowest category (OR: 0.57, 95% CI: 0.43, 0.75). The RCS analysis further supported a linear association between aMED scores and chronic constipation (Figure 2A). Additionally, higher consumption of vegetables, nuts, whole grains, and MUFA/SFA within the MED diet was significantly associated with a reduced likelihood of chronic constipation (Table S2).

**TABLE 2:** Association between aMED score and chronic constipation.

	Model 1^a^		Model 2^b^		Model 3^c^	
	OR (95% CI)	*p*	OR (95% CI)	*p*	OR (95% CI)	*p*
Continuous	0.83 (0.76, 0.89)	< 0.001	0.81 (0.75, 0.88)	< 0.001	0.82 (0.75, 0.89)	< 0.001
Categories						
Low (0–2)	Reference		Reference		Reference	
Moderate (3)	0.77 (0.62, 0.96)	0.023	0.74 (0.59, 0.92)	0.011	0.76 (0.61, 0.95)	0.022
High (4–9)	0.58 (0.45, 0.74)	< 0.001	0.55 (0.43, 0.70)	< 0.001	0.57 (0.43, 0.75)	< 0.001
*p* for trend		< 0.001		< 0.001		< 0.001

Abbreviations: aMED, alternative Mediterranean diet; CI, confidence interval; OR, odds ratio.

^a^ Model 1: adjusted for no covariates.

^b^ Model 2: adjusted for age, gender, and race.

^c^ Model 3: adjusted for age, gender, race, education, PIR, BMI, smoking, physical activity, hypertension, diabetes, and major depression.

### 3.3 Association Between MED Adherence and Chronic Diarrhea

Table 3 summarizes the relationship between aMED scores and chronic diarrhea. In the partially adjusted model, a unit increase in aMED score was linked to reduced odds of chronic diarrhea (OR: 0.89, 95% CI: 0.81, 0.98). However, this association did not persist in the fully adjusted model (OR: 0.97, 95% CI: 0.87, 1.07). When evaluated by categories, individuals in the highest aMED scores category exhibited lower odds of chronic diarrhea compared to those in the lowest category in the partially adjusted model (OR: 0.71, 95% CI: 0.53, 0.95), yet this association was not statistically significant in the fully adjusted model **(OR: 0.90, 95% CI: 0.66, 1.23)**. The RCS analysis provided no evidence of a nonlinear relationship between aMED scores and chronic diarrhea (Figure 2B). Moreover, none of the individual components of the MED diet were significantly associated with chronic diarrhea (Table S3).

**TABLE 3:** Association between aMED score and chronic diarrhea.

	Model 1^a^		Model 2^b^		Model 3^c^	
	OR (95% CI)	*p*	OR (95% CI)	*p*	OR (95% CI)	*p*
Continuous	0.92 (0.84, 1.00)	0.060	0.89 (0.81, 0.98)	0.022	0.97 (0.87, 1.07)	0.492
Categories						
Low (0–2)	Reference		Reference		Reference	
Moderate (3)	0.90 (0.72, 1.14)	0.396	0.86 (0.68, 1.09)	0.219	0.95 (0.74, 1.22)	0.653
High (4–9)	0.77 (0.59, 1.01)	0.069	0.71 (0.53, 0.95)	0.027	0.90 (0.66, 1.23)	0.491
*p* for trend		0.067		0.026		0.492

Abbreviations: aMED, alternative Mediterranean diet; CI, confidence interval; OR, odds ratio.

^a^ Model 1: adjusted for no covariates.

^b^ Model 2: adjusted for age, gender, and race.

^c^ Model 3: adjusted for age, gender, race, education, PIR, BMI, smoking, physical activity, hypertension, diabetes, and major depression.

### 3.4 Subgroup Analysis

**FIGURE 3:**
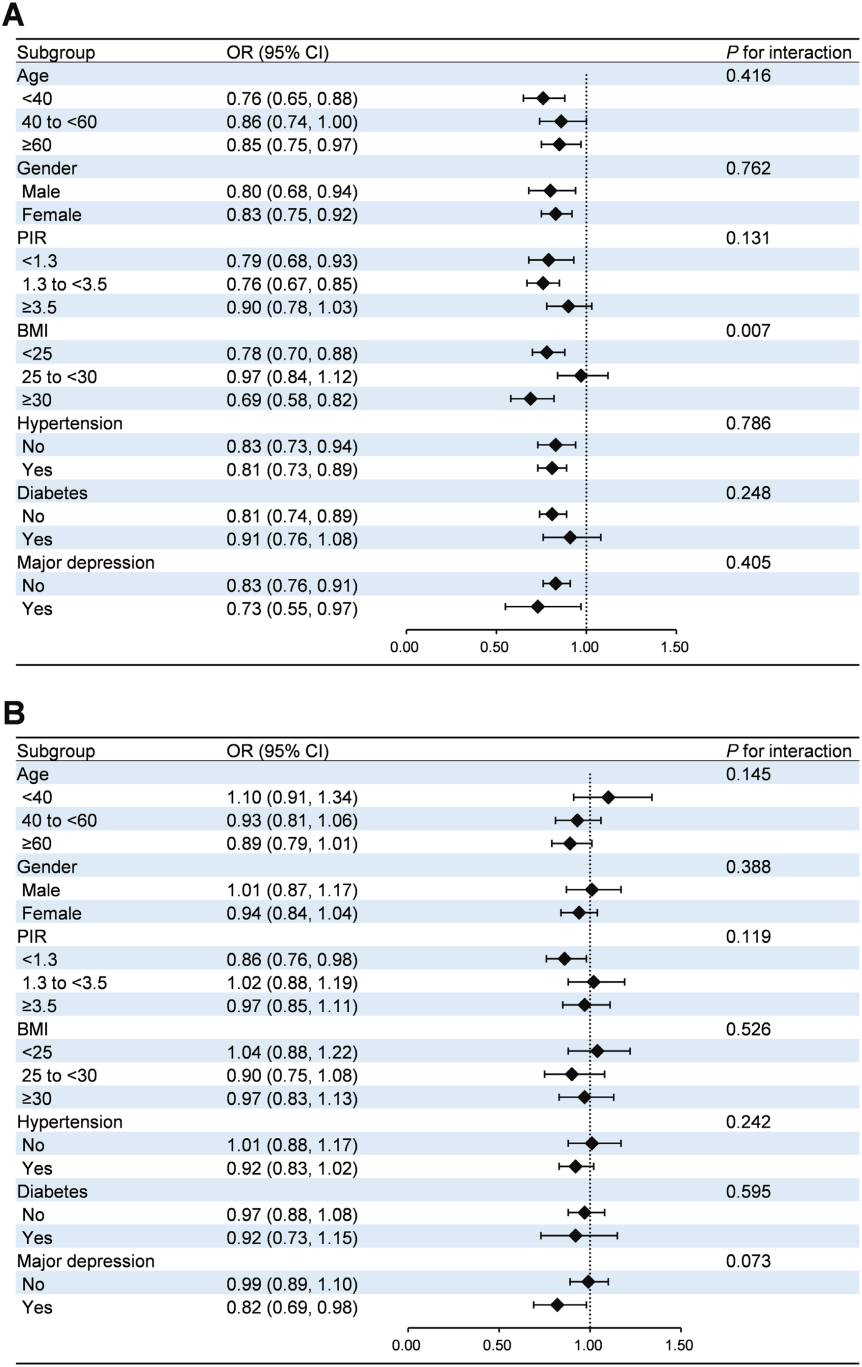
Subgroup analysis for the association between aMED score and abnormal bowel habits. (A) Chronic constipation; (B) Chronic diarrhea. The model was adjusted for age, gender, race, education, PIR, BMI, smoking, physical activity, hypertension, diabetes, and major depression, except for the stratification variable itself. BMI, body mass index; PIR, poverty income ratio.

Subgroup analyses and interaction tests were performed to determine if the relationships between aMED scores and chronic constipation or chronic diarrhea varied by age, gender, PIR, BMI, hypertension, diabetes, and major depression. For chronic constipation, the inverse relationship with aMED scores was most pronounced among obese individuals (OR: 0.69, 95% CI: 0.58, 0.82), with a significant interaction identified (Figure 3A). Conversely, no significant subgroup interactions were detected for the association between aMED scores and chronic diarrhea (Figure 3B).

## 4 Discussion

In this large, nationally representative study of U.S. adults, adherence to the MED was inversely associated with chronic constipation, with the strongest associations observed among individuals with obesity. No significant associations were identified between MED adherence and chronic diarrhea.

Diet is widely recognized as a key factor influencing functional gastrointestinal disorders (Singh et al. 2022). Emerging evidence supports beneficial effects of the MED on gastrointestinal health. Higher adherence to the MED has been inversely associated with the prevalence of functional gastrointestinal disorders (Agakidis et al. 2019; Zito et al. 2016), though some studies have reported no significant link between the MED and symptom severity (Altomare et al. 2021; Chen et al. 2024). Previous studies have also shown that MED adherence is associated with reduced constipation symptoms in individuals with Parkinson's disease (Bisaglia 2022; Seelarbokus et al. 2024), suggesting possible benefits of this dietary pattern for bowel function. Despite these findings, there is still a lack of data on the relationship between MED adherence and bowel habits in the general population.

Our findings contribute to this evidence base by demonstrating a significant inverse association between MED adherence and chronic constipation in a nationally representative sample of U.S. adults. Subgroup analyses indicate that this association is strongest among individuals with obesity. Previous research has established a positive relationship between obesity‐related indices and higher rates of constipation (Silveira et al. 2021; Xiang et al. 2024). Simultaneously, MED has been consistently identified as an effective dietary approach for managing obesity and improving metabolic health (Muscogiuri et al. 2022). These results suggest that the MED may reduce the prevalence of constipation through pathways related to improvements in metabolic health.

The observed inverse association between MED adherence and chronic constipation may be explained through several interconnected mechanisms. The high fiber content inherent in MED serves as a cornerstone for its beneficial effects on bowel regularity (Haro et al. 2016; Timm et al. 2023). Insoluble fibers increase fecal bulk and accelerate colonic transit by mechanically stimulating intestinal mechanoreceptors, while soluble fibers form gel‐like substances that soften stools and ease passage (Bijkerk et al. 2004; Gill et al. 2021). Notably, the fermentation of fiber by gut microbiota is a critical step in unlocking the full potential of the MED for improved colonic function (Gamrath et al. 2025). This process generates SCFAs, which act as the primary energy source for colonocytes and directly stimulate colonic motility (Holscher 2017; Zhang et al. 2023). Furthermore, adherence to the MED has been shown to positively influence the gut microbiota, increasing populations of beneficial bacteria such as *Bifidobacteria* and *Lactobacilli*, which are involved in SCFAs production and help maintain gut homeostasis (Illescas et al. 2021; Liu et al. 2025; Tan et al. 2023). Additionally, the high intake of MUFAs, predominantly derived from olive oil, nuts, and fish, modulates eicosanoid production, thereby exerting anti‐inflammatory effects (Djuric et al. 2015). Reduced intestinal inflammation enhances gut barrier integrity, which in turn facilitates normal colonic motility and alleviates constipation (Merra et al. 2020).

In contrast, this study did not observe a significant association between adherence to MED and chronic diarrhea, consistent with previous findings in individuals with Parkinson's disease (Seelarbokus et al. 2024). Future research should systematically investigate the effects of MED on chronic diarrhea across diverse populations and health conditions, considering the heterogeneous etiologies of gastrointestinal symptoms and variability in individual dietary responses (Dinu et al. 2018). Furthermore, longitudinal studies are essential to elucidate whether sustained MED adherence promotes long‐term gastrointestinal health, offering valuable evidence to inform the development of personalized dietary interventions.

Our study used a large, nationally representative sample of the U.S. population and accounted for a broad range of confounding factors, which enhances the generalizability and robustness of our results. Nevertheless, several limitations warrant consideration. The cross‐sectional nature of the NHANES design precludes causal inferences regarding the relationship between MED adherence and abnormal bowel habits. Reverse causality is possible, as preexisting gastrointestinal conditions might influence dietary choices. Future large‐scale prospective cohort studies are needed to clarify these relationships. Moreover, while the BSFS is a valid tool for classifying stool consistency, its reliance on a single self‐reported assessment may not fully capture long‐term bowel habits in participants. Finally, certain covariates were excluded from the analysis due to the unavailability of data, such as the use of medications that could affect bowel habits.

## 5 Conclusion

In conclusion, our findings show an inverse association between MED adherence and chronic constipation, while no significant relationship was observed between MED adherence and chronic diarrhea in the U.S. population. These findings reveal the potential role of the MED in promoting gastrointestinal health. However, further longitudinal studies are necessary to establish causal relationships and identify specific populations that may benefit most from MED adherence.

## Author Contributions

**Cheng Xu:** conceptualization, methodology, formal analysis, investigation, validation, writing – original draft. **Yu‐han Lin:** software, investigation, validation, visualization. **Xing‐chi Yu:** software, data curation, visualization. **Gai‐bo Huang:** data curation, visualization. **Jing‐yi Hu:** supervision. **Hong Shen:** supervision, writing – review and editing. **Chong‐chao Li:** supervision, validation, funding acquisition, writing – review and editing. All authors contributed to the article and approved the submitted version.

## Ethics Statement

The National Center for Health Statistics Research Ethics Review Board provided ethics approval (Protocol #2005‐06) for all study protocols in the NHANES, and written informed consent was obtained from all participants. Therefore, no external ethical approval or informed consent were required.

## Consent

The authors have nothing to report.

## Conflicts of Interest

The authors declare no conflicts of interest.

## Supporting information


**Table S1:** fsn370809‐sup‐0001‐Tables.pdf.

## Data Availability

The National Health and Nutrition Examination Survey dataset is publicly available at the National Center for Health Statistics of the Centers for Disease Control and Prevention (https://www.cdc.gov/nchs/nhanes/index.htm).
