# Supplementary material for: Association of Mediterranean Diet Adherence With Chronic Constipation and Chronic Diarrhea: Findings From NHANES
Source: Food Sci Nutr. 2025 Aug 13;13(8):e70809. doi: 10.1002/fsn3.70809 (PMC12351127; doi:10.1002/fsn3.70809)
Supplement: Supplementary file 1 — Table S1: fsn370809‐sup‐0001‐Tables.pdf. [file FSN3-13-e70809-s001.pdf]

Table S1 Components and scoring criteria of aMED.

Component	Food	Score of 0	score of 1
1.Vegetables	Coleslaw, Side salad, Avocado, Beetroot, Broccoli, Butternut squash, Cabbage/kale, Carrot, Cauliflower, Celery, Courgette, Cucumber, Garlic, Leek, Lettuce, Mushroom, Onion, Parsnip, Sweet pepper, Spinach, Other vegetables	For each component, intakes below the median score 0	For each component, intakes above the median score 1
2.Legumes	Baked bean, Pulses, Broad bean, Tofu		
3.Fruits	Fruit juice, Stewed fruit, Prune, Dried fruit, Mixed fruit, Apple, Banana, Berry, Cherry, Grape, Mango, Melon, Orange, Satsuma, Other fruit		
4.Nuts	Salted peanuts, Unsalted peanuts, Salted nuts, Unsalted nuts, Seeds		
5.Whole grains	Muesli, Oat crunch, Sweetened cereal, Plain cereal, Bran cereal, Whole-wheat cereal, Other cereal, Oatcakes, Wholemeal pasta, Brown rice, Couscous, Cereal bar		
6.Fish	Tinned tuna, Oily fish, Breaded fish, Battered fish, White fish, Prawns, Lobster/crab, Shellfish, Other fish		
7. MUFA/SFA	Monounsaturated fatty acids, Saturated fatty acids		
8.Red and processed meat	Sausage, Beef, Pork, Lamb, Bacon, Ham, Liver	For each component, intakes above the median score 0	For each component, intakes below the median score 1
9.Alcohol	Alcohol	<10 g/d or > 25 g/d for men or < 5 g/d or > 15 g/d for men = 0	10-25 g/day for men or 5-15 g/day for women =1
Total score		0	9

Abbreviations: aMED, alternative Mediterranean diet; MUFA, monounsaturated fatty acids; SFA, saturated fatty acids.

Table S2 Associations of individual food components of MED with chronic constipation.

	OR (95% CI)	*P* value
Vegetables		**<0.001**
<median	Reference	
≥median	0.57 (0.46, 0.71)	
Legumes		0.339
<median	Reference	
≥median	0.84 (0.59, 1.19)	
Fruits		0.119
<median	Reference	
≥median	0.81 (0.63, 1.05)	
Nuts		**0.047**
<median	Reference	
≥median	0.76 (0.59, 0.98)	
Whole grains		**0.029**
<median	Reference	
≥median	0.73 (0.55, 0.95)	
Fish		0.191
<median	Reference	
≥median	0.77 (0.53, 1.13)	
MUFA/SFA		**0.009**
<median	Reference	
≥median	0.78 (0.65, 0.93)	
Red and processed meat		0.352
<median	Reference	
≥median	1.14 (0.87, 1.51)	
Moderate Alcohol consumption		0.204
No	Reference	
Yes	0.68 (0.39, 1.21)	

Abbreviations: MED, Mediterranean diet; MUFA, monounsaturated fatty acids; SFA, saturated fatty acids; OR, odds ratio; CI, confidence interval.

Table S3 Associations of individual food components of MED with chronic diarrhea.

	OR (95% CI)	*P* value
Vegetables		0.084
<median	Reference	
≥median	0.78 (0.59, 1.04)	
Legumes		0.573
<median	Reference	
≥median	1.08 (0.83, 1.42)	
Fruits		0.440
<median	Reference	
≥median	0.90 (0.69, 1.18)	
Nuts		0.399
<median	Reference	
≥median	0.89 (0.68, 1.16)	
Whole grains		0.051
<median	Reference	
≥median	0.73 (0.53, 1.00)	
Fish		0.100
<median	Reference	
≥median	1.23 (0.96, 1.58)	
MUFA/SFA		0.071
<median	Reference	
≥median	1.33 (0.99, 1.78)	
Red and processed meat		0.286
<median	Reference	
≥median	1.17 (0.88, 1.54)	
Moderate Alcohol consumption		0.362
No	Reference	
Yes	0.76 (0.42, 1.37)	

Abbreviations: MED, Mediterranean diet; MUFA, monounsaturated fatty acids; SFA, saturated fatty acids; OR, odds ratio; CI, confidence interval.
